# Proteasome Inhibitor Bortezomib Ameliorates Intestinal Injury in Mice

**DOI:** 10.1371/journal.pone.0034587

**Published:** 2012-03-30

**Authors:** Koichi Yanaba, Yoshihide Asano, Yayoi Tada, Makoto Sugaya, Takafumi Kadono, Shinichi Sato

**Affiliations:** Department of Dermatology, Faculty of Medicine, University of Tokyo, Tokyo, Japan; University of Ottawa, Canada

## Abstract

**Background:**

Bortezomib is a proteasome inhibitor that has shown impressive efficacy in the treatment of multiple myeloma. In mice, the addition of dextran sulfate sodium (DSS) to drinking water leads to acute colitis that can serve as an experimental animal model for human ulcerative colitis.

**Methodology/Principal Findings:**

Bortezomib treatment was shown to potently inhibit murine DSS-induced colitis. The attenuation of DSS-induced colitis was associated with decreased inflammatory cell infiltration in the colon. Specifically, bortezomib-treated mice showed significantly decreased numbers of CD4^+^ and CD8^+^ T cells in the colon and mesenteric lymph nodes. Bortezomib treatment significantly diminished interferon (IFN)-γ expression in the colon and mesenteric lymph nodes. Furthermore, cytoplasmic IFN-γ production by CD4^+^ and CD8^+^ T cells in mesenteric lymph nodes was substantially decreased by bortezomib treatment. Notably, bortezomib enhanced T cell apoptosis by inhibiting nuclear factor-κB activation during DSS-induced colitis.

**Conclusions/Significance:**

Bortezomib treatment is likely to induce T cell death, thereby suppressing DSS-induced colitis by reducing IFN-γ production.

## Introduction

Ulcerative colitis is an inflammatory bowel disease characterized by pathologic mucosal damage and ulceration, which can involve the rectum and extend proximally [1]. Although its etiology and pathogenesis have not yet been identified, inappropriate activation of the mucosal immune system has been found to play an important role in mucosal inflammation. At sites of intestinal inflammation, granulocytes and macrophages produce high levels of pro-inflammatory cytokines, including interleukin (IL)-1β, IL-6, and tumor necrosis factor (TNF)-α [2], [3], which are directly involved in the pathogenesis of ulcerative colitis.

The oral administration of dextran sulfate sodium (DSS) solution to rodents is widely employed as a model of human ulcerative colitis, because it causes acute inflammatory reactions and ulceration in the entire colon similar to that observed in patients [4], [5]. Mice exposed to DSS in drinking water develop inflammation only in the large intestine and show signs such as diarrhea, hematochezia, and body weight loss with histologic findings including inflammatory cell infiltration, erosion, ulceration, and crypt abscesses. Furthermore, increased production of pro-inflammatory cytokines, including interferon (IFN)- γ, TNF-α, IL-1, IL-6, IL-12, and IL-17, has been found in the colon of mice with DSS-induced colitis [6], [7].

The major intracellular pathway for protein degradation is the ubiquitin-proteasome pathway [8]. Proteasomes are large multimeric protease complexes located in both the cytoplasm and nuclei that selectively and timely degrade most cellular proteins [9], [10]. The 26S proteasome consists of a central 20S core and two 19S regulatory complexes. Upon stimulation, the formation of immunoproteasomes is induced. The ubiquitination of target proteins is an important mechanism for the discriminatory nature of protein degradation by proteasomes [10]. Proteasome inhibitors have received much attention because of their potent anti-tumor activity [11]. In particular, bortezomib, a boronic acid dipeptide derivative, is a specific protease inhibitor that has recently been approved for the treatment of relapsed multiple myeloma, a plasma cell neoplasia, because of its direct growth-inhibitory and apoptotic effects on this cancer [11], [12]. Furthermore, bortezomib is effective in the treatment of allograft rejection, graft-versus-host disease, contact hypersensitivity responses, and lupus-like disease in mice [13]–[16]. Proteasome inhibitors induce apoptosis in activated and proliferating, but not resting, T cells [17], [18], suggesting one possible mechanism for the suppression of T cell-mediated immune responses by bortezomib. In this study, the effect of bortezomib in ulcerative colitis was examined using DSS-induced mouse colitis.

## Results

### Bortezomib treatment attenuates DSS-induced colitis

To assess the therapeutic effect of bortezomib in DSS-induced colitis, we treated mice with 3% DSS for 7 days and quantitatively evaluated the severity of intestinal injury by measuring body weight and disease activity index (DAI) scores. We treated mice twice weekly with bortezomib or phosphate buffered saline (PBS) control starting 2 days before DSS administration. DAI scores were based on weight loss, stool consistency, and bleeding.

**Figure 1:**
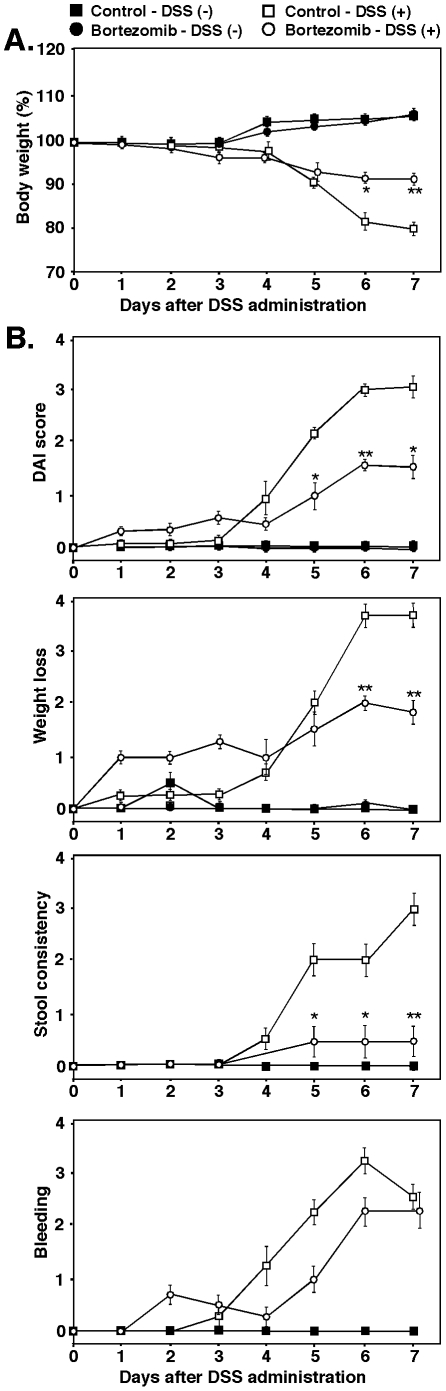
Bortezomib suppressed the severity of DSS-induced colitis. Mice ingested either DSS solution or normal drinking water. Mice were treated with 200 µl of bortezomib (0.75 mg/kg) or PBS (control) intravenously twice weekly, starting 2 days prior to DSS administration. The severity of intestinal injury was evaluated by quantitatively measuring body weight (**A**) and DAI scores (**B**). DAI scores were based on weight loss, stool consistency, and bleeding. Values represent means (±SEM) from ≥4 mice per group. Significant differences between sample means are indicated: *P<0.05; **P<0.01. Similar results were obtained in at least two independent experiments.

Statistically significant body weight loss was first observed in DSS-treated mice on day 6 (Figure 1A). Bortezomib treatment significantly attenuated body weight loss compared with control-treated mice and delayed the increase in DAI scores by 1 day from day 4 (control-treated mice) to day 5 (bortezomib-treated mice) (Figure 1B). DAI scores were also significantly higher in bortezomib-treated mice than in control-treated mice from day 5–7. Each element of the DAI score showed the same trend as the overall DAI score, suggesting that bortezomib treatment suppressed DSS-induced colitis in mice.

**Figure 2:**
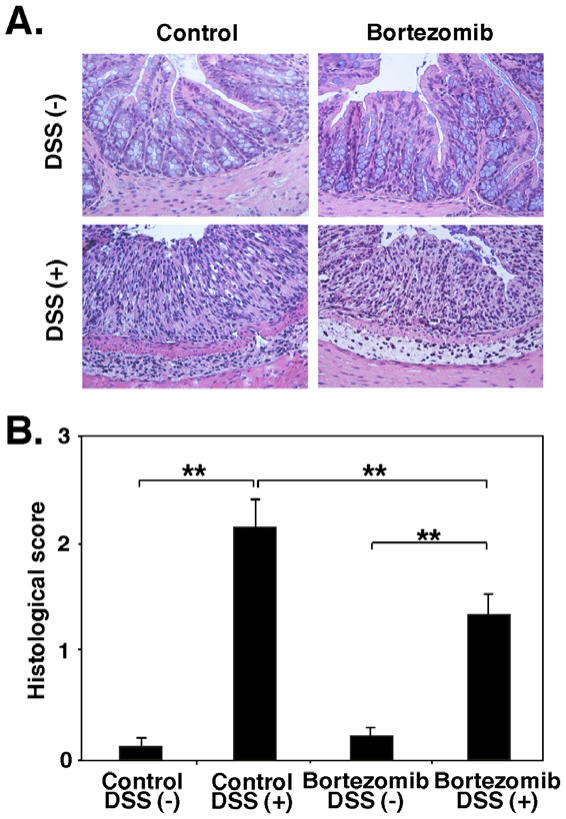
Bortezomib treatment reduced the severity of DSS-induced colitis. Colon sections were harvested from mice treated with bortezomib or control after ingestion of either DSS solution or normal drinking water for 7 days, and stained with hematoxylin and eosin. (**A**) Representative colon sections at 7 days after induction of colitis. (**B**) Histologic sections were blindly scored on a scale of 0–4 for severity of colitis. Values represent means (±SEM) from ≥4 mice per group. Significant differences between sample means are indicated: **P<0.01**. Results represent one of two independent experiments producing similar results.

To further evaluate disease severity, the degree of intestinal injury was assessed histopathologically. Following the 7-day period of ingestion of 3% DSS or normal drinking water, colons were removed for histopathologic evaluation (Figure 2A). DSS treatment induced epithelial injury and increased mononuclear cell infiltration and inflammatory changes in submucosal tissues of control-treated mice, but these changes were less severe in bortezomib-treated mice. The pathologic scores were significantly lower in bortezomib-treated mice than in control-treated mice (*P*<0.01; Figure 2B). Thus, bortezomib treatment reduced DSS-induced colitis both clinically and histopathologically.

### Bortezomib inhibits T cell accumulation during DSS-induced colitis

**Figure 3:**
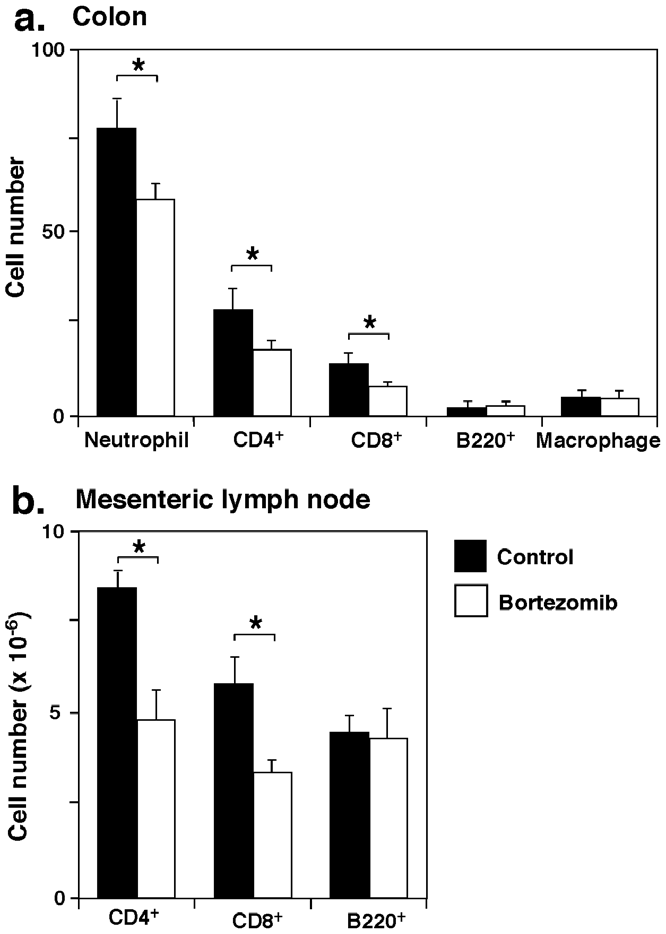
Profile of infiltrating cells in the colon and mesenteric lymph nodes during DSS-induced colitis in mice treated with bortezomib or control. (**A**) The numbers of neutrophils, CD4^+^ T cells, CD8^+^ T cells, B220^+^ B cells, and F4/80^+^ macrophages per one field of view (×200) in the colon were counted. (**B**) Bortezomib affects CD4^+^ and CD8^+^ T cell numbers in mesenteric lymph nodes during DSS-induced colitis. Bar graphs indicate CD4^+^ T cells, CD8^+^ T cells, and B220^+^ cells in mesenteric lymph nodes 7 days after induction of colitis. A, B) Values represent means (± SEM) from ≥4 mice per group. Significant differences between sample means are indicated: *P<0.05. Similar results were obtained in at least two independent experiments.

The profile of infiltrating cells in the colon was further examined immunohistologically. Seven days after DSS administration, neutrophil numbers were significantly decreased in bortezomib-treated mice relative to control-treated mice (23% decrease, *P*<0.05; Figure 3A). The numbers of CD4^+^ and CD8^+^ T cells were also significantly lower in mice treated with bortezomib than in control mice (38% decrease, *P*<0.05 and 41% decrease, *P*<0.05, respectively), but there were no significant differences in numbers of B cells or macrophages. Thus, bortezomib treatment reduced both neutrophil and T cell infiltration in the colon.

To determine whether bortezomib treatment altered the populations of T cells and B cells in mesenteric lymph nodes during DSS-induced colitis, the numbers of CD4^+^, CD8^+^, and B220^+^ cells in mesenteric lymph nodes were assessed by flow cytometry 7 days after DSS administration. Mice treated with bortezomib had fewer CD4^+^ and CD8^+^ T cells than control mice (42% decrease, *P*<0.05 and 41% decrease, *P*<0.05, respectively; Figure 3B). Thus, bortezomib treatment decreased the numbers of both CD4^+^ and CD8^+^ T cells in mesenteric lymph nodes.

### Bortezomib reduces IFN-γ production during DSS-induced colitis

**Figure 4:**
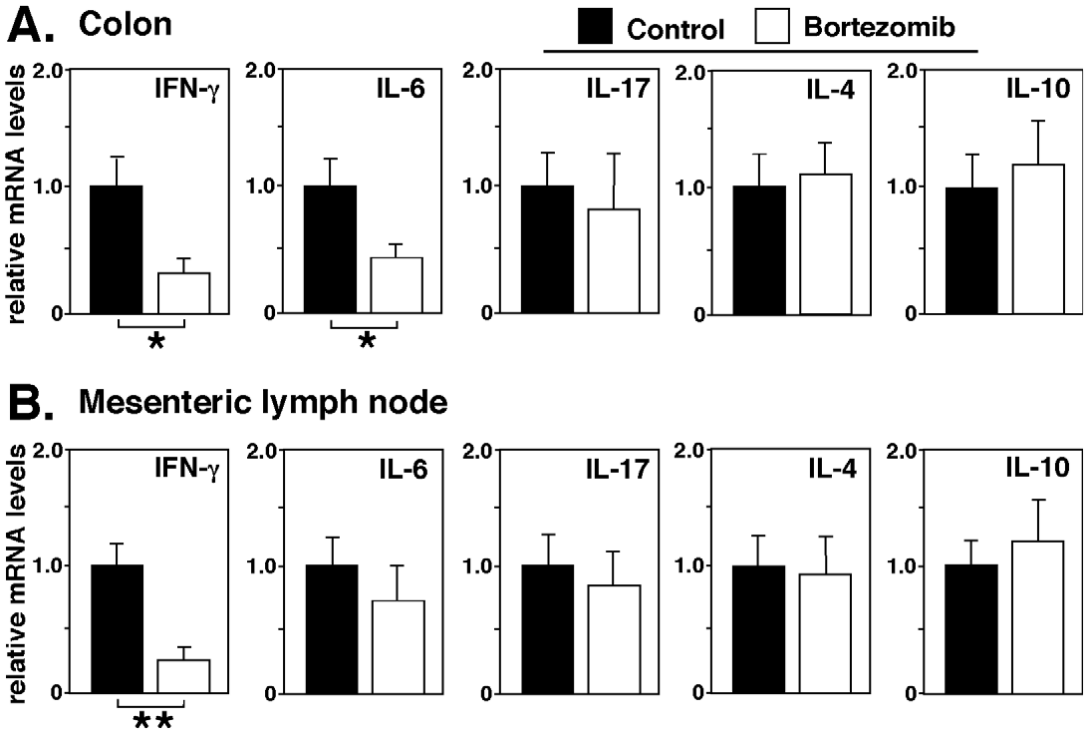
Bortezomib treatment affects cytokine production in DSS-induced colits. Cytokine mRNA expression in the colon (**A**) and mesenteric lymph nodes (**B**) in mice treated with bortezomib or control during DSS-induced colitis. Colon tissue and mesenteric lymph nodes were collected from control- or bortezomib-treated mice 7 days following DSS administration. Transcript levels were quantified by real-time PCR analysis and were normalized with an internal control. Values represent means (± SEM) from ≥4 mice of each group. Significant differences between sample means are indicated; *P<0.05, **P<0.01. Results represent one of two independent experiments producing similar results.

The effect of bortezomib treatment on cytokine expression during DSS-induced colitis was examined by assessing the mRNA expression of several cytokines in control and bortezomib-treated mice. Colons and mesenteric lymph nodes were harvested 7 days after the induction of colitis, and the expression of IFN-γ, IL-6, IL-17, IL-4, and IL-10 mRNA was quantified by real-time PCR. Bortezomib treatment significantly decreased IFN-γ colon mRNA levels by 70% and IL-6 colon mRNA levels by 58% compared with control mice (*P*<0.05, Figure 4A). By contrast, mRNA expression of IL-17, IL-4 and IL-10 in the colons was not affected by bortezomib treatment. In mesenteric lymph nodes, IFN-γ transcripts of bortezomib-treated mice were significantly decreased relative to those of control mice (75% decrease, *P*<0.01; Figure 4B). IL-6, IL-17, IL-4 and IL-10 mRNA expression in mice treated with bortezomib was comparable to control mice. Thus, bortezomib treatment substantially decreased IFN-γ mRNA levels during DSS-induced colitis.

### Bortezomib diminishes T cell IFN-γ production during DSS-induced colitis

**Figure 5:**
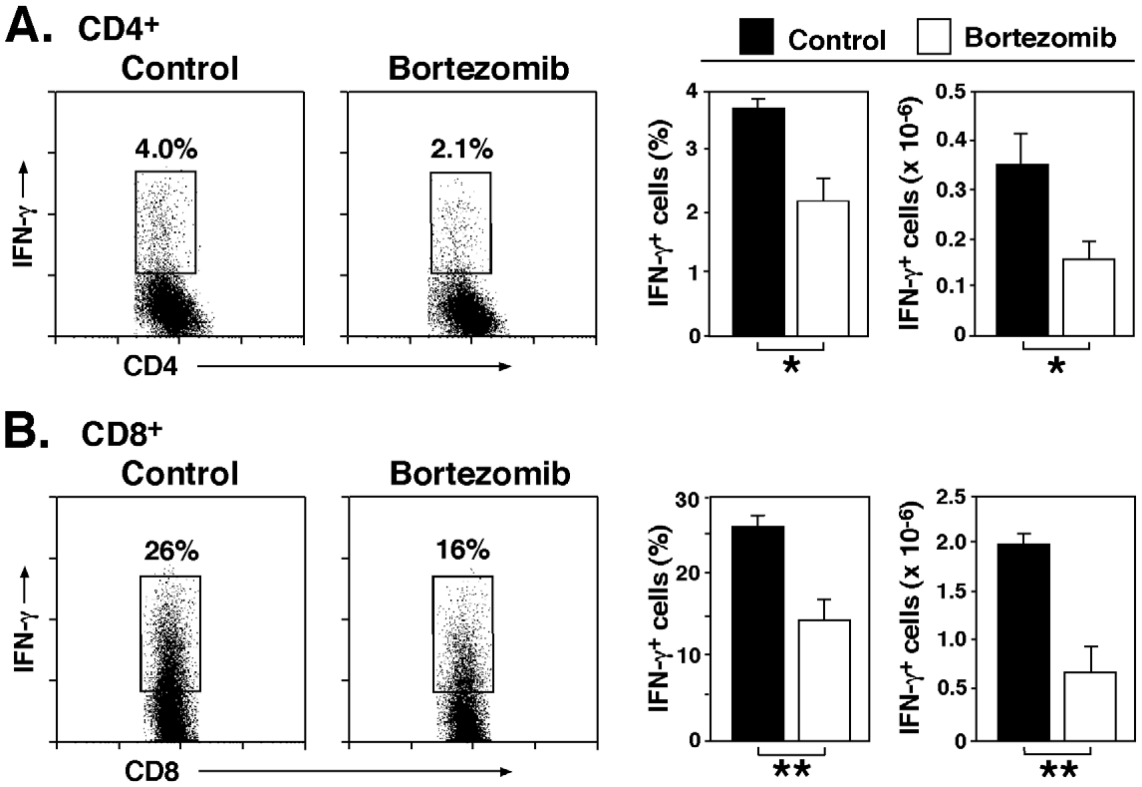
Bortezomib treatment reduces IFN-γ production by CD4^+^ and CD8^+^ T cells in mesenteric lymph nodes during DSS-induced colitis. IFN-γ production by mesenteric lymph node CD4^+^ (**A**) or CD8^+^ (**B**) T cells 7 days following DSS administration as determined by intracellular cytokine staining with flow cytometry analysis. Bar graphs indicate mean (± SEM) percentages and numbers of draining lymph node IFN-γ-producing CD4^+^ or CD8^+^ T cells following bortezomib or control treatment in one representative experiment with three mice per group. Significant differences between PBS-treated mice versus other group are indicated; *P<0.05, **P<0.01. Similar results were obtained in at least two independent experiments.

Intracellular cytokine staining was used to assess whether bortezomib treatment affected IFN-γ production from mesenteric lymph node CD4^+^ and CD8^+^ T cells. Bortezomib treatment significantly reduced the frequency (43% decrease, *P*<0.05) and number (53% decrease, *P*<0.01) of IFN-γ-producing CD4^+^ T cells compared with control mice (Figure 5A). Furthermore, the frequency and number of CD8^+^ T cells in mice treated with bortezomib were 40% (*P*<0.05) and 66% (*P*<0.01) lower, respectively, than in control mice (Figure 5B). Thus, bortezomib treatment diminished T cell IFN-γ production during DSS-induced colitis.

### Bortezomib induces T cell apoptosis by inhibiting NF-κB activation during DSS-induced colitis

**Figure 6:**
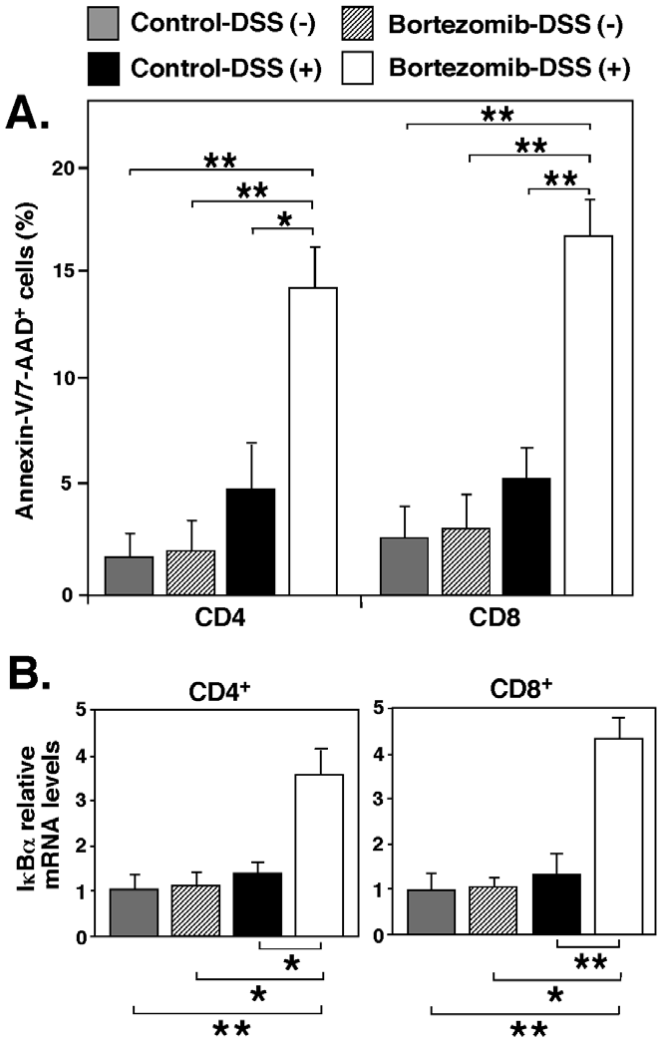
Bortezomib treatment enhances T cell apoptosis by inhibiting NF-κB activation. (**A**) The percentages of Annexin-V/7-AAD^+^ CD4^+^ and CD8^+^ T cells in mesenteric lymph nodes 7 days after the administration of DSS. Bar graphs indicate mean (± SEM) percentages of mesenteric lymph node Annexin-V/7-AAD^+^ CD4^+^ and CD8^+^ T cells following bortezomib or control treatment in one representative experiment with 4 mice per group. (**B**) IκBα mRNA expression in mesenteric lymph nodes CD4^+^ and CD8^+^ T cells. Transcript levels were quantified by real-time PCR analysis and were normalized with an internal control. Values represent means (± SEM) from ≥4 mice of each group. A, B) Significant differences between sample means are indicated; *P<0.05; **P<0.01. Similar results were obtained in at least two independent experiments.

To determine whether bortezomib treatment induced T cell apoptosis, thereby suppressing DSS-induced colitis, the percentages of Annexin-V/7-AAD^+^ CD4^+^ and CD8^+^ T cells in mesenteric lymph nodes were assessed 7 days after the induction of colitis. In mice treated with bortezomib, the numbers of Annexin-V/7-AAD^+^ CD4^+^ and CD8^+^ T cells were significantly increased compared with control-treated mice (2.7-fold, *P*<0.05 and 3.2-fold, *P*<0.01, respectively; Figure 6A). As proteasome inhibitors counteract NF-κB activation by interfering with the proteasomal degradation of IκB proteins, we also assessed the transcriptional activity of NF-κB using real-time PCR for NF-κB-induced IκBα mRNA. IκB mRNA levels of CD4^+^ and CD8^+^ T cells in mesenteric lymph nodes from bortezomib-treated mice were significantly enhanced compared with control-treated mice (2.5-fold, *P*<0.05 and 3.2-fold, *P*<0.01, respectively; Figure 6B). Thus, NF-κB inhibition is likely to contribute to bortezomib-induced cell death in T cells, thereby suppressing DSS-induced colitis.

## Discussion

The results of this study demonstrate that bortezomib treatment inhibits DSS-induced colitis in mice (Figures 1 and 2). The suppression of DSS-induced colitis by bortezomib treatment correlated with a decrease in CD4^+^ and CD8^+^ T cell accumulation both in the colon and mesenteric lymph nodes (Figure 3). Remarkably, bortezomib treatment significantly reduced IFN-γ mRNA expression in the colon and mesenteric lymph nodes, while IL-17, IL-4 and IL10 mRNA expression was not affected (Figure 4). Consistently, IFN-γ production from CD4^+^ and CD8^+^ T cells in mesenteric lymph nodes was inhibited by bortezomib treatment (Figure 5). Thus, bortezomib treatment attenuated DSS-induced colitis by inhibiting excessive IFN-γ production from CD4^+^ and CD8^+^ T cells.

The immunoproteasome subunit LMP7 is critical for proteasome activity [19], and it was recently reported that LMP7-deficiency is associated with reduced severity of DSS-induced colitis in mice [20]. Patients with inflammatory bowel disease exhibit high levels of LMP7 in the inflamed gut [21], and their increased proteasome activity induced by high levels of expression of immunoproteasome subunits mediates sustained activation of NF-κB [22]. These results suggest that proteasome inhibitors may ameliorate inflammatory bowel disease. In this study, bortezomib administration substantially reduced the severity of DSS-induced colitis as well as significantly enhancing apoptosis and IκB expression of CD4^+^ and CD8^+^ T cells during DSS-induced colitis (Figure 6). Thus, NF-κB inhibition is likely to contribute to bortezomib-induced cell death in T cells, thereby suppressing DSS-induced colitis.

Bortezomib treatment of mice with lupus-like disease significantly improves the disease severity by reducing the numbers of both CD4^+^ and CD8^+^ T cells in the spleen [14]. Bortezomib treatment also demonstrates significant protection from acute graft-versus-host disease in a murine allogeneic bone marrow transplantation model by inhibiting allogeneic T cell proliferation [16]. By contrast, bortezomib administration largely eliminates plasma cells but not T cells or B cells in murine models of human systemic lupus erythematosus [14]. In the current study, bortezomib treatment reduced the numbers of CD4^+^ and CD8^+^ T cells, but not B cells or macrophages during DSS-induced colitis. It has been reported that proliferating T cells are more sensitive to bortezomib-mediated cytotoxity than resting T cells [16], [17]. Proteasome inhibitors induce endoplasmic reticulum stress-induced apoptosis in multiple myeloma cells as a result of the terminal unfolded protein response [23], while inhibition of proteasome activities by proteasome inhibitors induces apoptosis preferentially in rapid proliferating neoplastic cells [24]. Thus, bortezomib treatment is likely to eliminate only excessively proliferating immune cells, thereby suppressing harmful inflammatory responses.

Proteasome inhibition using bortezomib has recently emerged as an effective anticancer therapy [11]. Thus far, the therapeutic feasibility of protease inhibition in inflammatory and autoimmune diseases has been revealed only in murine models of human systemic lupus erythematosus [14], experimental autoimmune encephalomyelitis [25], [26], rheumatoid arthritis [27], asthma [28], and contact dermatitis [13]. In the current study, bortezomib treatment in mice resulted in attenuated DSS-induced colitis, suggesting that bortezomib may also be effective for the treatment of human ulcerative colitis. Patients with this disease are generally treated with anti-inflammatory and immunosuppressive drugs, antizbiotics, and biologics such as anti-tumor necrosis factor therapies and/or surgery. However, such therapies do not cure the disease and patients suffer a life-long illness. Further studies are needed to determine the precise mechanisms by which bortezomib treatment reduces the severity of DSS-induced colitis. Nonetheless, if the efficacy seen in mice translates to humans, the current results may provide new insights and therapeutic approaches for treating ulcerative colitis.

## Materials and Methods

### Ethics statement

All animal studies and procedures were approved by The Committee on Animal Experimentation of the University of Tokyo, Tokyo, Japan (Approval ID: P10-134).

### Induction and evaluation of DSS-induced colitis

C57BL/6 mice were purchased from Clea Japan Inc. (Tokyo, Japan), bred in a pathogen-free barrier facility and used at 8–12 weeks of age. Three % (w/v) DSS (molecular mass, 36–50 kDa; Sigma, St. Louis, MO) was dissolved in purified water and administered to mice instead of normal drinking water for 7 days [29]. The volume of water intake was measured daily to determine the amount of DSS consumed per mouse; this was comparable between treatment groups in all experiments. To analyze the therapeutic effect of bortezomib (LC Laboratories, Woburn, MA), 0.75 mg/kg was injected in 200 µl PBS through lateral tail veins twice weekly, starting 2 days before the administration of DSS. Control animals received an identical volume of PBS alone.

Clinical DAI scoring for DSS-induced colitis was based on weight loss, stool consistency, and bleeding as described previously [30]. The DAI was scored on a scale from 0–4 for each clinical parameter and then averaged for each group. Weight changes were based on the starting weight of each mouse at the initiation of DSS treatment. Weight-loss scores were determined as follows: 0, no weight loss; 1, 1–5% weight loss; 2, 6–10% weight loss; 3, 11–15% weight loss; and 4, >15% weight loss. Stool samples were collected from each mouse at all timepoints and stool scores were determined as follows: 0, normal stools; 2, loose stools; and 4, diarrhea. Fecal blood testing kits (Shionogi, Osaka, Japan) were used to check the stools for the presence of blood. Bleeding scores were determined as: 0, no bleeding; 1, guiaiac occult blood test (minimal color change to green); 2, guiaiac occult blood test (maximal color change to blue); 3, blood visibly present in the stool and no clotting on the anus; and 4, gross bleeding from the anus with clotting present.

### Histologic analysis

Mice were sacrificed 5 days after the induction of intestinal injury. Colon samples were removed and segments fixed in 10% buffered formalin. After paraffin embedding, 5-µm-thick sections were cut and stained with hematoxylin and eosin. Histologic scoring was based on a previously described method [29]. Briefly, hematoxylin and eosin-stained cross-sections of the descending colon tissue were scored microscopically in a blinded fashion on a scale from 0–4 based on the following histologic criteria: 0, no change from normal tissue; 1, low level of inflammation with scattered infiltrating mononuclear cells (1–2 foci); 2, moderate inflammation with multiple foci; 3, high level of inflammation with increased vascular density and marked wall thickening; 4, maximal severity of inflammation with transmural leukocyte infiltration and loss of goblet cells. An average of four fields of view per colon were evaluated for each mouse. These scores were averaged for each group and recorded as the histopathology score.

For immunohistochemistry, frozen tissue sections of the colon samples were acetone-fixed and incubated with 10% normal rabbit serum in PBS for 10 minutes at 37°C to block non-specific staining. Sections were then incubated with rat mAbs specific for mouse CD4, CD8, B220 (BD PharMingen; San Diego, CA), and macrophages (F4/80; American Type Culture Collection; Rockville, MD). Rat IgG (Southern Biotechnology Associates Inc., Birmingham, AL) was used as a control for non-specific staining. Sections were then incubated sequentially for 20 minutes at 37°C with a biotinylated rabbit anti-rat IgG and then horseradish peroxidase-conjugated avidin–biotin complex (Vectastain ABC kit; Vector Laboratories, Burlingame, CA). Sections were developed with 3,3′-diaminobenzidine tetrahydrochloride and hydrogen peroxide and counterstained with methyl green. Stained cells were counted in 10 random grids under high-magnification (×400) power fields of a light microscope. Each section was examined independently by two investigators in a blinded manner.

### Cell isolation

Single-cell suspensions of spleen and mesenteric lymph nodes were generated by gentle dissection. Magnetic cell sorting technology (Miltenyi Biotech, Auburn, CA) was used to purify CD4^+^ and CD8^+^ T cell lymphocyte populations.

### Antibodies and immunofluorescence analysis

Anti-mouse mAbs B220 (RA3-6B2), CD4 (H129.19), CD8 (53-6.7), and CD19 (1D3) were from BD PharMingen. Intracellular staining used mAbs reactive with IFN-γ (XMG1.2), IL-17A (eBio17B7), and IL-4 (BVD6-24G2), and the Cytofix/Cytoperm kit (BD Biosciences, San Diego, CA). Single cell suspensions of draining lymph nodes (paired axillary ad inguinal) were generated by gentle dissection. Viable cells were counted using a hemocytometer, with relative lymphocyte percentages determined by flow cytometry analysis. Single-cell leukocyte suspensions were stained on ice using predetermined optimal concentrations of each antibody for 20–60 minutes, and fixed as described [31]. For intracellular cytokine staining, lymphocytes were stimulated *in vitro* with phorbol 12-myristate 13-acetate (50 ng/ml; Sigma-Aldrich), ionomycin 1 µg/ml; Sigma-Aldrich) in the presence of monensin (1 µM; eBioscience) for 5 hours before staining. In certain experiments, apoptosis was measured by flow-cytometric staining for Annexin-V and 7-AAD (BD PharMingen). Cells with lymphocyte light scatter properties were analyzed by 2–4 color immunofluorescence staining and FACSCalibur flow cytometers (Becton Dickinson, San Jose, CA). Background staining was determined using unreactive isotype-matched control mAbs (Caltag Laboratories, San Francisco, CA) with gates positioned to exclude ≥98% of unreactive cells.

### RNA isolation and real-time reverse transcription-PCR

Total RNA was isolated from colon specimens and mesenteric lymph node suspensions with RNeasy spin columns (Qiagen, Crawley, UK). Total RNA from each sample was reverse-transcribed into cDNA. Expression of IL-4, IL-6, IL-10, IL-17, IFN-γ and IκBα was analyzed using a real-time PCR quantification method according to the manufacturer's instructions (Applied Biosystems, Foster City, CA). Sequence-specific primers and probes were designed by Pre-Developed TaqMan assay reagents or Assay-On-Demand (Applied Biosystems). Real-time PCR (40 cycles of denaturing at 92°C for 15 seconds and annealing at 60°C for 60 seconds) was performed on an ABI Prism 7000 sequence detector (Applied Biosystems). Glyceraldehyde-3-phosphate was used to normalize mRNA. Relative expression of real-time PCR products was determined by using ΔΔCT methods [32] to compare target gene and housekeeping gene mRNA expression. One of the control samples was chosen as a calibrator sample.

### Statistical analysis

All data are expressed as mean ± SEM. The Mann–Whitney U test was used to determine the level of significance of differences in sample means, and the Bonferroni test was used for multiple comparisons.
